# High Serum PD-L1 Levels Are Associated with Poor Survival in Urothelial Cancer Patients Treated with Chemotherapy and Immune Checkpoint Inhibitor Therapy

**DOI:** 10.3390/cancers13112548

**Published:** 2021-05-22

**Authors:** Ulrich Krafft, Csilla Olah, Henning Reis, Claudia Kesch, Christopher Darr, Viktor Grünwald, Stephan Tschirdewahn, Boris Hadaschik, Orsolya Horvath, Istvan Kenessey, Peter Nyirady, Melinda Varadi, Orsolya Modos, Anita Csizmarik, Tibor Szarvas

**Affiliations:** 1 West German Cancer Center, Department of Urology, University of Duisburg-Essen, 45147 Essen, Germany; Ulrich.Krafft@uk-essen.de (U.K.); Csilla.Olah@uk-essen.de (C.O.); Claudia.Kesch@uk-essen.de (C.K.); Christopher.Darr@uk-essen.de (C.D.); Stephan.Tschirdewahn@uk-essen.de (S.T.); Boris.Hadaschik@uk-essen.de (B.H.); 2 Institute of Pathology, University of Duisburg-Essen, 45147 Essen, Germany; Henning.Reis@uk-essen.de; 3 Clinic for Urology and Clinic for Medical Oncology, West German Cancer Center, University Hospital Essen, 45147 Essen, Germany; Viktor.Gruenwald@uk-essen.de; 4 Department of Genitourinary Medical Oncology and Pharmacology, National Institute of Oncology, 1122 Budapest, Hungary; horvath.orsolya@oncol.hu; 5 2nd Department of Pathology, Semmelweis University, 1122 Budapest, Hungary; kenessey.istvan@oncol.hu; 6 National Cancer Registry and Centre for Biostatistics, National Institute of Oncology, 1122 Budapest, Hungary; 7 Department of Urology, Semmelweis University, 1089 Budapest, Hungary; nyirady.peter@med.semmelweis-univ.hu (P.N.); varadi.melinda_rita@med.semmelweis-univ.hu (M.V.); modos.orsolya@med.semmelweis-univ.hu (O.M.); csizmarik.anita@med.semmelweis-univ.hu (A.C.)

**Keywords:** sPD-L1, bladder cancer, resistance, chemotherapy, immunotherapy

## Abstract

**Simple Summary:**

Advanced urothelial bladder cancer (BC) shows a heterogeneous response to both platinum and immune checkpoint inhibitor (ICI) therapies. The PD-1/PD-L1 signaling pathway represents an immune escape mechanism and tissue PD-L1 expression was shown to be associated with patients’ prognosis and therapy response in various solid tumors. In the present study, we found for the first time that higher pretreatment serum PD-L1 levels are associated with shorter survival in platinum- and ICI-treated BC patients.

**Abstract:**

Serum PD-L1 (sPD-L1) levels are associated with prognosis in various tumors but has not yet been investigated in advanced bladder cancer. We assessed pretreatment serum samples from 83 BC patients who received platinum chemotherapy and from 12 patients who underwent immune checkpoint inhibitor (ICI) therapy. In addition, on-treatment samples from further therapy cycles were collected during chemotherapy (*n* = 58) and ICI therapy (*n* = 11). Serum PD-L1 levels were determined using ELISA. High baseline sPD-L1 levels were associated with worse ECOG status (*p* = 0.007) and shorter overall survival for both chemotherapy- and ICI-treated patients (*p* = 0.002 and *p* = 0.040, respectively). Multivariate analysis revealed high baseline sPD-L1 level as an independent predictor of poor survival for platinum-treated patients (*p* = 0.002). A correlation analysis between serum concentrations of PD-L1 and matrix metalloprotease-7 (MMP-7)—a protease which was recently found to cleave PD-L1—revealed a positive correlation (*p* = 0.001). No significant sPD-L1 changes were detected during chemotherapy, while in contrast we found a strong, 25-fold increase in sPD-L1 levels during atezolizumab treatment. In conclusion, our work demonstrates that pretreatment sPD-L1 levels are associated with a poor prognosis of BC patients undergoing platinum and ICI therapy. Future research should prospectively address the value of sPD-L1 in predicting treatment response.

## 1. Introduction

Urothelial bladder cancer is the most common malignancy of the urinary tract and represents one of the most frequently diagnosed cancers in western countries [1]. Bladder cancers are clinically classified into non-muscle-invasive (NMIBC) and muscle-invasive bladder cancer (MIBC), with distinct implications for patient management. Radical cystectomy (RC) is the standard treatment for patients with MIBC. However, RC only provides 5-year survival in about 50% of patients [2]. To improve these results, cisplatin-based neoadjuvant chemotherapy (NAC) is recommended to eligible patients [3]. Nevertheless, only less than 20% of all patients receive NAC before RC [4]. Adjuvant platinum-based chemotherapy is administered after RC for T3/4 and/or lymph node metastatic tumors when no neoadjuvant treatment was applied. First-line platinum therapy for metastatic disease provides a median survival of ~15 months and a 5-year overall survival rate of ~15% [5]. In the last few years, novel ICI therapies (e.g., atezolizumab, pembrolizumab, nivolumab) became available for patients with urothelial carcinoma that had progressed during or following platinum-containing chemotherapy and for maintenance therapy after response to platinum-based chemotherapy (avelumab) [6,7]. These therapies provide 15–29% objective response rates, which are significantly higher than those of <10% for other second-line chemotherapies. Most importantly, responding patients benefited by a durable response of over 12 months, which is a never-before-seen improvement at this stage. In 2017, atezolizumab and pembrolizumab drugs were approved for the first-line setting in platinum ineligible patients [8]. In 2018, restrictions were made for the first-line treatment with atezolizumab and pembrolizumab as only patients with high PD-L1 immunostaining had better survival on ICI therapy compared to chemotherapy in the first-line setting [9]. These results warrant that only well-selected patients benefit from these novel therapies.

PD-L1 is an immune checkpoint molecule that modulates T-cell receptor signals and plays a major role in tumor immunity escape. Higher PD-L1 tumor tissue protein expression was shown to be associated with higher response rates and improved survival under ICI therapy. In addition, tissue expression of PD-L1 proved to be of prognostic in chemotherapy-treated MIBC patients [10]. Matrix metalloproteinases are able to proteolytically cleave the extracellular domain of PD-L1 leading to its functional loss [11] and increased levels of soluble PD-L1 in the extracellular space. More recently, the soluble forms of PD-L1 have been detected in the blood of patients with various malignancies and these were associated with patients’ outcome [12,13,14]. Based on these, we hypothesized that determination of serum PD-L1 may have prognostic value in MIBC patients who receive systemic therapies.

To our knowledge, sPD-L1 has not been analyzed in serum samples of MIBC patients yet. Therefore, we aimed to elucidate the prognostic role of serum PD-L1 levels in chemotherapy and immunotherapy-treated MIBC patients. In this explorative study, we used serum samples of an unselected, prospectively collected, real-life cohort of patients with progressed bladder cancer who underwent systemic platinum-based or ICI treatment. Our aim was to assess the prognostic value of pretreatment and on-treatment serum PD-L1 levels in these patients.

In addition, we formerly found significantly elevated urine and serum MMP-7 levels in MIBC patients present with lymph node or distant metastasis and higher pretreatment serum MMP-7 levels were significantly correlated with poor survival in platinum-treated MIBC patients [15,16,17]. As MMP-7 was shown to be capable of cleaving the ectodomain region of PD-L1 [18] and thereby may increase its levels in the blood circulation, we seek correlation of formerly determined serum MMP-7 concentrations and those of recently measured PD-L1 levels in the same serum samples. 

## 2. Materials and Methods

### 2.1. Patients

Pretreatment serum samples were collected from 83 MIBC patients (64 males and 19 females) who received neoadjuvant (*n* = 22), adjuvant (*n* = 33), or palliative (*n* = 28) platinum therapy between 01/2014 and 05/2019 at the Department of Urology at Semmelweis University. On-treatment samples taken before the 2nd or 3rd therapy cycle were available for 58 of 83 patients. Patients’ median age was 67 years ranging from 41 to 81. Sixty-one patients received gemcitabine/cisplatin chemotherapy while 22 patients were treated with gemcitabine/carboplatin. Adjuvant chemotherapy was applied within 90 days following curative radical cystectomy with curative intent. Neoadjuvant chemotherapy included three cycles of treatment and was started 3.7 months (median) prior radical cystectomy. Palliative chemotherapy was applied for those patients who were not eligible or denied radical cystectomy and for those who had distant metastases before platinum treatment (Table S1). Additionally, for 5 of 22 patients who received neoadjuvant chemotherapy pre- and postoperative serum samples were available.

In addition, pretreatment serum samples were drawn from 12 urothelial cancer patients (10 males, 2 females), who received ICI therapy (atezolizumab (*n* = 11) and pembrolizumab (*n* = 1)) either in 2nd line setting (*n* = 9) or in case of platinum-ineligibility (*n* = 3). Samples were collected between 4/2019 and 3/2020 at the Department of Urology, Semmelweis University. The vast majority (11/12) of ICI treatments were applied in the second line setting and one patient received neoadjuvant ICI therapy. The median time between start of chemotherapy and start of ICI therapy was 10.2 months. On-treatment samples before the 2nd immunotherapy cycle were available for 11 of 12 patients. The median time between the cystectomy and on-treatment sample collection was 4.9 months and the time between the cystectomy and last treatment before the follow-up sample collection was 3.9 months. The median age of these patients was 69 years ranging from 63 to 77. Patients’ characteristics are given in Table 1 and in Table S1.

In 21 cases, formalin-fixed paraffin-embedded (FFPE) tissue samples were available for PD-L1 immunohistochemical analysis. 

Age, gender, ECOG (Eastern Cooperative Oncology Group), stage, LN status, and M status (soft tissue, bone) were noted. Overall survival (OS) was calculated as the time from first cycle of chemo- or immunotherapy (baseline sample) to death. The study was conducted in accordance with the Helsinki Declaration and approved by the institutional ethics committee (TUKEB 55/2014 and 224/2013). Written consent of all patients was available.

### 2.2. Serum PD-L1 Enzyme-Linked Immunosorbent Assay (ELISA) 

Serum PD-L1 levels were determined using the PD-L1/B7-H1 Quantikine ELISA kit (DB7H10, R&D Systems, Wiesbaden, Germany) in accordance with the manufacturer’s instructions. The cut-off value of PD-L1 for dichotomization was set at the median (83 pg/mL) and at upper 25 percentile (103 pg/mL) for the chemotherapy cohort and 90 pg/mL (median) for the ICI treated cohort. Serum MMP-7 levels were measured using the Quantikine ELISA kit from R&D Systems (Cat.Nr.: DMP700) and published in previous work of our group [17]. To exclude a possible interference/cross reactivity between the therapeutic anti-PD-L1 antibody (atezolizumab, Tecentriq®, Roche, Basel, Suisse) and our ELISA kit, we performed an ELISA analysis with atezolizumab and pembrolizumab.

### 2.3. Tissue PD-L1 Immunohistochemical Analysis

PD-L1 IHC analysis was carried on 3μm thick FFPE tissue sections from 21 available tumor tissues on a Ventana Benchmark Ultra system (Ventana Medical Systems, Tucson, AZ, USA). PD-L1 analyses were performed using clone 22C3 (M3653, Agilent/DAKO, Carpinteria, CA, USA). PD-L1 expression was determined for immune cells (IC-Score) and combined with tumor cells (CPS, combined positive score) as follows: IC/Immune cell Score: 0  =  < 1%, 1  =  1– < 5%, 2  =  5– < 10%, 3 =  > 10%, and the combined positivity score (CPS) given by summing the number of PD-L1–stained cells (tumor cells, immune cells) and dividing the result by the total number of viable tumor cells, multiplied by 100 with an upper limit of 100. 

### 2.4. Statistical Analysis 

The Kaplan–Meier log-rank test and univariate Cox analysis were performed for univariate survival analysis. In multiple Cox analyses, parameters with a *p*-value of less than 0.05 were included in the multivariate analysis. In all tests, *p* < 0.05 was considered an indication of statistical significance. Spearman’s rank correlation analysis was used to test for correlation between serum MMP-7 and PD-L1 and between serum and tissue levels of PD-L1. All statistical analyses were performed with the software package IBM SPSS Statistics for Windows (Version 27.0. Armonk, NY, USA: IBM Corp).

## 3. Results

### 3.1. Clinical Background 

Patients’ characteristics are given in Table 1. In the chemotherapy cohort, 59 of 83 patients, and in the ICI cohort, 8 of 12 patients underwent prior cystectomy. The median number of therapy cycles was three (range: one–nine) in the chemotherapy cohort and five (range: 2–17) in the ICI cohort. The median follow-up time was 14 months for the chemotherapy cohort with 45 (54%) deaths, while the median follow-up time was 17 months for the ICI cohort with 4 (33%) deaths. 

### 3.2. Correlation of Serum PD-L1 Concentrations with Clinicopathological Parameters and Survival

For the chemotherapy cohort, pretreatment serum PD-L1 values showed no association with age, gender and lymphatic or visceral metastasis, while worse ECOG status was associated with higher sPD-L1 level (*p* = 0.007) (Table 2). In the subgroup of patients who received neoadjuvant chemotherapy significantly higher baseline sPD-L1 values were found in high stage (T3–4) compared to stage T0-T2 patients (*p* = 0.001) (Table S2). For the ICI cohort low number of samples did not allow a valid statistical analysis. 

Our study did not include non-cancerous controls; however, the producer of the assay provided reference values from 36 healthy individuals (62.5 pg/mL range: 44.5–106) that are lower compared to the concentrations we found in the chemotherapy (83.0 pg/mL, range: 0–781) and ICI cohorts (90.0 pg/mL, range: 25.3–169.0). In addition, using the same ELISA kit, Chen et al. reported that the median serum PD-L1 level in 21 healthy controls was 48.2 pg/mL [19]. However, a direct comparison is not possible in our study, serum PD-L1 levels in bladder cancer seems to be higher as in non-tumorous controls.

As former studies reported a reverse association between serum PD-L1 concentrations and adverse events (AE), we assessed this aspect in our ICI cohort; 3 of 12 immunotherapy patients experienced treatment-related adverse events. Of these, two patients experienced grade 1 adverse events (AE) and one patient developed a grade 3 adverse event. The patient with a grade 3 AE showed a distinct lower sPD-L1 (25.28 pg/mL) compared to the two patients with grade 1 AEs (95.19 pg/mL and 58.98 pg/mL) and to those without AEs (96.21 pg/m).

**Figure 1:**
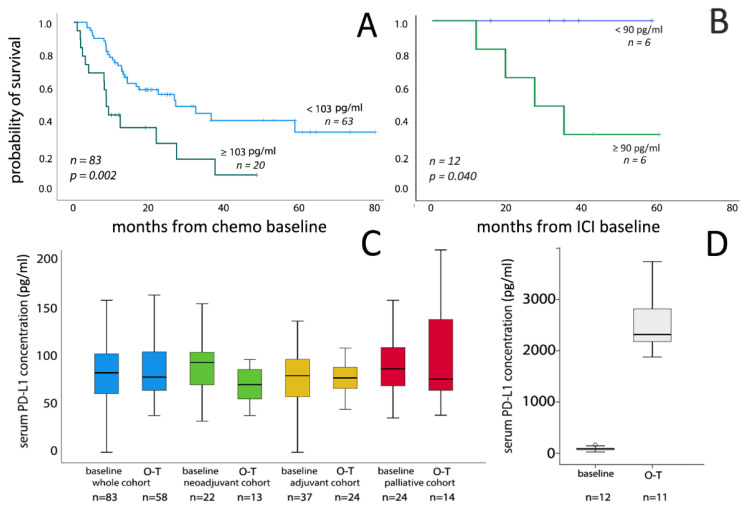
Overall survival stratified by pretreatment sPD-L1 levels in the chemotherapy (**A**) and ICI-treated patients (**B**). Box-plot presentation of serum PD-L1 levels in pretreatment and follow-up samples (collected at 2-3. therapy cycles) in platinum-treated (**C**) and immune checkpoint inhibitor-treated (**D**) bladder cancer patients. Please note the strong increase of sPD-L1 levels in on-treatment (O-T) serum samples taken during ICI therapy.

Univariate analysis revealed a significant association between high pretreatment serum PD-L1 values and shorter OS for the whole chemotherapy cohort (*p* = 0.002) (Figure 1A, Table 3). This association could be observed in the subgroups of adjuvant and palliative treatment groups (*p* = 0.007 and *p* = 0.012, respectively), while it was not present in the neoadjuvant treatment group and when sPD-L1 was included as a continuous variable (Table S3). Univariate analysis showed that sPD-L1 levels were prognostic both in the distant metastatic and in metastasis-free subgroups (Figure S1). Multivariate analysis demonstrated that high baseline serum PD-L1 levels >103 pg/mL, presence of metastases and ECOG (>0) status were independent predictors of shorter OS for the whole chemotherapy cohort (*p* = 0.002, *p* = 0.002 and *p* < 0.001, respectively; Table 3). This was also the case when the median sPD-L1 levels was used for dichotomization (HR: 1.843; 95% CI 1.012–3.357; *p* = 0.046). Similarly, in ICI treated patients higher pretreatment serum PD-L1 concentrations were significantly associated with poor OS in univariate analysis both as a dichotomized (*p* = 0.040) and continuous (*p* = 0.045) variable (Figure 1B). We performed a Cox analysis for the whole study cohort (chemo- and ICI-treated patients, *n* = 95). Only ECOG status (>0) and sPD-L1 concentrations proved to be associated with patients’ survival. The median cut-off value for the whole study cohort was 85 pg/mL. Cox univariate survival analysis demonstrated that patients with sPD-L1 levels above the median had significantly shorter OS (HR: 2.171, 95%CI: 1.989–3.600, *p* = 0.023). Similarly, PD-L1 serum levels proved to be prognostic when applied as a continuous variable for the whole study cohort (HR: 1.003, 95%CI: 1.001–1.005, *p* = 0.006). In the multivariate analysis, ECOG performance status (>0) and sPD-L1 above 85 pg/mL concentration remained significant (*p* = 0.012), while sPD-L1 as continuous variable slightly missed the significance level (*p* = 0.056). 

### 3.3. Serum PD-L1 Level Changes during Chemotherapy

We detected no significant difference between baseline and on-treatment serum PD-L1 levels in the whole chemotherapy cohort (Figure 1C). Similarly, subgroup analysis did not show significant changes of serum PD-L1 levels in the adjuvant and palliative treatment groups. In contrast, in patients who received neoadjuvant chemotherapy serum PD-L1 levels significantly decreased in the on-treatment samples of platinum therapy (*p* = 0.029). 

### 3.4. Serum PD-L1 Level Changes during ICI Therapy

For 11 of 12 patients in the ICI group, on-treatment samples were available. Interestingly, we found a >25-fold increase in serum PD-L1 concentrations in all on-treatment samples after atezolizumab treatment (Figure 1D), while no such increase could be observed in the pembrolizumab-treated patient. To exclude a possible interference/cross reactivity between the therapeutic anti-PD-L1 antibody (atezolizumab) and our ELISA kit, we performed an ELISA analysis with atezolizumab solution which showed no positive color reaction in our ELISA assay, suggesting no interference between the therapeutic anti-PD-L1 antibody and the antibodies used in the ELISA.

### 3.5. Tissue PD-L1 Expression and Its Corrrelation with Serum PD-L1 Levels

PD-L1 immunohistochemistry showed the typical staining characteristics known from routine use with membranous and/or cytoplasmic staining in tumor cells and/or immune cells and a range from negative to weak, moderate, and strong staining intensities. Non-specific staining in necrotic tissue was disregarded from analyses. Spearman correlation analysis revealed no significant correlation between tissue expression and serum concentration levels of PD-L1 (rs = 0.127, *p* = 0.603). 

### 3.6. Correlation Between Serum PD-L1 and MMP-7 Concentrations

As MMP-7 has been shown to be able to proteolytically cleave membrane bound PD-L1 and we formerly measured serum MMP-7 concentrations in samples of patients treated with adjuvant chemotherapy (*n* = 23) [17]. Here, we performed the Spearmen´s rank correlation analysis between sPD-L1 and MMP7, which revealed a significant positive correlation between the concentrations of these two proteins (rs = 0.525, *p* = 0.001) (Figure S2).

## 4. Discussion

Patients with advanced urothelial carcinoma show a heterogeneous response to both platinum-based chemotherapy and ICI therapy. In the last years, many attempts have been made to identify clinical and molecular factors that are able to predict the response to chemotherapy as well as to ICI therapy with the aim of improving therapeutic decision-making. Gene expression based molecular subtype classification systems have been suggested for MIBC with potential prognostic and predictive implications [20]. However, the therapy predictive value of molecular subtype classifications still needs to be validated. Among other mutations in DNA repair mechanisms such as *ERCC2* as well as increased tissue expression of ERCC1, Emmprin, and Survivin were identified as potential chemotherapy-predictive markers [21,22]. Furthermore, the IMvigor210 trial revealed a significant correlation between tumor mutational burden (TMB) and higher response rates and improved OS in ICI treated MIBC patients [23]. Currently, however, the only routinely applied method for the prediction of ICI therapy is the immunohistochemical determination of tissue PD-L1 status [6]. Tissue expression of PD-L1 was also found to be of prognostic value for chemotherapy-treated MIBC patients and PD-L1 has been suggested to influence chemotherapy resistance [10,24]. Therefore, we hypothesized that determination of serum PD-L1 may have prognostic value in MIBC patients who receive systemic therapies. Serum PD-L1 levels have already been investigated in various malignancies. In gastrointestinal cancers, higher pretreatment serum PD-L1 levels were found to be associated with shorter OS and PFS in chemotherapy treated patients [25,26]. Similarly, in non-small-cell lung cancer (NSCLC), high serum PD-L1 levels independently correlated with poor outcomes for patients who received chemotherapy as well as for those who were treated with ICI therapy. Interestingly, Costantini and colleagues found no prognostic value for serum PD-L1 at baseline but high PD-L1 levels (>33.97 pg/mL) in on-treatment samples taken at first tumor evaluation were associated with poor response to ICI therapy and a shorter PFS and OS [12]. Furthermore, for renal cell cancer, sPD-L1 was found to be elevated in patients compared to healthy controls and in tyrosine kinase inhibitor-treated patients, high baseline sPD-L1 level was associated with poor OS [13,14]. In line with the aforementioned works, in the present study we found a significant and independent correlation of high sPD-L1 levels with poor OS in chemotherapy-treated MIBC patients. This correlation, however, was different in various treatment groups; showing the strongest association in the adjuvant treatment group followed by the palliative treatment group, while this correlation was proved not significant in the neoadjuvant treatment group. Similar to the chemotherapy-treated patients, high pretreatment serum PD-L1 levels were associated with shorter OS in ICI-treated patients. These findings are in accordance with the observations made in NSCLC, where higher PD-L1 serum levels were associated with poor outcome in ICI-treated patients [12]. Interestingly, the unfavorable prognostic effect of high serum PD-L1 levels in ICI-treated MIBC patients seems to oppose its favorable predictive value when measured in tissue samples. This suggest that high serum PD-L1 levels probably do not directly reflect its expression levels in the tumor tissue. Accordingly, our correlation analysis of tissue vs. corresponding serum PD-L1 levels found no significant association. Similarly, no association was discovered between tumor PD-L1 expression (by IHC) and serum PD-L1 concentrations (by ELISA) in patients with diffuse large B-cell lymphomas and renal cell cancers, suggesting that tumor microenvironment, may also be involved in the regulation of serum PD-L1 levels [27,28]. As recently demonstrated, increased serum PD-L1 might be generated as a result of a proteolytic degradation by MMP-7 [18]. Similarly, Aguiree et al. in Chron’s disease showed that increased MMP-7 expression was associated with decreased PD-L1 levels in intestinal mucosa cells. Addition of recombinant MMP-7 resulted in decreased PD-L1 levels and MMP-inhibition was able to reverse this effect [29]. Therefore, we speculate that high serum PD-L1 levels may be the consequence of their enhanced cleavage in tissue samples. In accordance with this hypothesis, we found a significant positive correlation between serum PD-L1 and MMP-7 levels. Therefore, an enhanced proteolytic milieu may lead to a decrease of tissue PD-L1 and therefore a lower sensitivity to ICI. Furthermore, the cleaved soluble extracellular domain of PD-L1 may exert inhibitory effect on immune cells or trap therapeutic antibodies leading to a decreased therapeutic effect [30].

Our results need to be validated in independent patient cohorts, focusing on those groups (adjuvant and palliative platinum and ICI treatment groups) in which sPD-L1 proved to be prognostic. On the other hand, as serum PD-L1 levels proved to be prognostic both in the chemotherapy and the ICI treatment cohorts, it seems to be a prognostic rather than a therapy predicting marker, therefore serum PD-L1 analysis may not be valuable for supporting therapeutic decision between chemotherapy and ICI-treatment in progressed cases of MIBC. However, patients with high sPD-L1 are at higher risk under both platinum and ICI therapy and might therefore benefit from other therapy modalities. Therefore, further research should answer the question whether locally progressed and/or metastatic MIBC patients with high sPD-L1 levels may better benefit from targeted therapies (e.g., anti-FGFR, nectin-4 targeted or the currently approved sacituzumab govitecan) [31,32].

Our analyses in follow-up samples of ICI-treated MIBC patients revealed strongly, 25-fold elevated serum PD-L1 levels in all patients after treatment with atezolizumab. Interestingly, no such increase could be observed in the pembrolizumab-treated patient and measurement of atezolizumab solution in our ELISA showed no positivity in the assay excluding the possibility of a cross-reactivity with the assay. A published dataset with available pre- and post-treatment PD-L1 expression in patients who received neoadjuvant atezolizumab therapy revealed a moderate but significant increase in PD-L1 expression in the post-treatment tissues, however this increase was much lower compared to that we found in the serum samples and therefore we suppose that bladder cancer cells may not be the primary source of the increased post-treatment serum PD-L1 flare-up [33]. Therefore, we suggest that immune cells might overexpress PD-L1 or a forced PD-L1 shedding may occur as a response to the atezolizumab treatment. However, further research is needed to clarify the biological background and the clinical relevance of the characteristic sPD-L1 flare-up that we observed in the present study.

## 5. Conclusions

In this study, we demonstrated, that pretreatment serum PD-L1 is independently associated with poor survival of platinum-treated bladder cancer patients. Similarly, high sPD-L1 levels were associated with shorter OS in ICI-treated patients. The opposite prognostic effects of serum and tissue PD-L1 suggests that serum PD-L1 levels does not reflect tissue expression but may rather be related to its increased proteolytic shedding. Our data suggest that MMP-7 may play a role in this proteolytic process. Finally, we found a strong flare-up of sPD-L1 levels following treatment with atezolizumab, which needs to be further investigated.

## Figures and Tables

**Table 1:** Patients’ characteristics.

Variables	Whole Chemo Cohort	ICI Cohort
	*n* (%)	*n* (%)
Total number of patients	83	12
Age at baseline median (range)	67 (41–81)	69 (63–77)
Gender		
male	64 (77)	10 (83)
female	19 (33)	2 (17)
ECOG PS at enrollment		
0	48 (58)	10 (84)
1	32 (37)	1 (8)
2	3 (3)	1 (8)
Cystectomy data		
Cystectomy performed	59 (71)	8 (67)
pT0	3 (5)	1 (8)
pT1	2 (3)	0 (0)
pT2	11 (19)	1 (8)
pT3	30 (51)	5 (42)
pT4	12 (20)	1 (8)
n.a.	1	4 (33)
R	10 (17)	0 (0)
LN metastasis at RC	30 (51)	5 (42)
Chemo baseline data		
LN at baseline	36 (43)	4 (33)
Distant metastasis at baseline	12 (15)	7 (58)
Soft tissue lesions (lung/liver)	9 (11)	5 (42)
Bone metastasis	3 (3)	2 (17)
Setting of chemotherapy		
neoadjuvant	22 (27)	-
adjuvant	33 (40)	-
palliative	28 (33)	-
Chemotherapy regimen		
Gem/Cis	61 (73)	-
Gem/Carbo	22 (27)	-
Atezolizumab	-	11 (92)
Pembrolizumab	-	1 (8)
Number of cycles median (range)	3 (1–9)	5 (2–17)
single (only one series)	9	0
Number of patients died (%)	45 (54)	4 (33)
Follow-up time in months median (range)	14 (1–80)	17 (6–31)
PD-L1 serum levels		
PD-L1 median (range) baseline [pg/mL]	83.0 (0.0–781)	90.0 (25.3–169.0)
PD-L1 median (range) 2–3 cycle [pg/mL]	78.5 (0.0–273.1)	2316 (42.5–3818)

ECOG PS—Eastern Cooperative Oncology Group performance status. LN—lymph node metastasis, R—positive surgical margin, RC—cystectomy, Gem/Cis—gemcitabine + cisplatin, Gem/Carbo—gemcitabine + carboplatin, ICI—immune checkpoint inhibitor, n.a.—not available.

**Table 2:** Correlation of pretreatment sPD-L1 levels (pg/mL) with clinicopathological parameters.

Variables		All Patients			Whole Chemo Cohort			ICI Cohort		
		*n*	Median (Range)	*p*	*n*	Median (Range)	*p*	*n*	Median (Range)	*p*
Age	≤65	38	84.4 (0.0–780.9)	0.566	35	82.2 (0.0–780.9)	0.430	3	86.6 (59.0–169.0)	-
	>65	57	84.9 (25.3–243.1)		48	84.0 (35.8–243.1)		9	93.3 (25.3–145.0)	
Gender	Male	74	86.1 (0.0–780.9)	0.950	64	84.0 (0.0–780.9)	0.435	10	94.2 (59.0–169.0)	-
	Female	10	82.2 (25.3–194.9)		19	82.2 (40.5–194.9)		2	26.1 (25.3–26.89)	
ECOG	0	58	80.8 (0.0–169.0)	**0.022**	48	80.4 (0.0–158.5)	**0.007**	10	91.9 (26.9–169.0)	-
	1–2	37	95.9 (25.3–780.9)		35	96.2 (35.8–780.9)		2	59.3 (25.3–93.3)	
Stage at cystectomy	Not performed	28			24			4		
	n.a.	1			1			0		
	pT0	4	92.2 (46.0–111.4)	0.792	3	97.7 (46.0–111.0)	0.858	1	86.6	-
	pT1	2	135.2 (75.6–194.9)		2	135.2 (75.6–194.9)		0	-	
	pT2	12	78.4 (26.9–154.9)		11	80.7 (32.4–154.9)		1	26.9	
	pT3	35	81.5 (25.8–324.0)		30	80.4 (25.8–324.0)		5	99.1 (59.0–169.0)	
	pT4	13	88.7 (0.0–158.5)		12	84.4 (0.0–158.5)		1	93.3	
	pT1–pT2	18	83.7 (26.9–194.9)	0.259	16	84.7 (32.4–194.9)	0.741	2	56.8 (26.9–86.6)	-
	pT3–pT4	48	85.1 (0.0–324.0)		42	80.4 (0.0–324.0)		6	97.2 (59.0–169.0)	
LN/M status	N0/M0	40	88.6 (25.8–324.0)	0.277	38	87.0 (38.5–324.0)	0.209	2	87.1 (80.9–93.3)	-
	N+ or M+	55	85.1 (0.0–158.8)		45	80.7 (0.0–780.9)		10	90.9 (25.3–169.0)	

ECOG—Eastern Cooperative Oncology Group performance status, LN—lymph node metastasis, M—distant metastasis, ICI—immune checkpoint inhibitor, n.a.—not available. In the ICI cohort, due to the small number of cases no Cox regression analysis could be performed. Bold printed *p*-values were significant (<0.05).

**Table 3:** Cox uni- and multivariate OS analysis in platinum-treated patients.

Variables			Univariate			Multivariate		
		*n*	HR	95% CI	*p*	HR	95% CI	*p*
Ag	≤65	35	ref.			-	-	-
	>65	48	1.297	0.716–2.351	0.391	-	-	-
Gender	Male	64	ref.			-	-	-
	Female	19	0.815	0.392–1.694	0.584	-	-	-
ECOG	0	48	ref.			ref.		
	1–2	35	2.615	1.381–4.950	**0.003**	3.692	1.870–7.287	**<0.001**
Stage at cystectomy *	T0–T2	16	ref.			-	-	-
	T3–T4	42	1.135	0.483–2.664	0.772	-	-	-
LN/M status *	N0/M0	38	ref.			ref.		
	N+ or M+	45	1.914	1.017–3.601	**0.044**	2.904	1.502–5.615	**0.002**
PD-L1 serum cc./upper 25%	<103 pg/mL	63	ref.			ref.		
	≥103 pg/mL	20	1.381	1.120–1.703	**0.002**	1.41	1.135–1.752	**0.002**
PD-L1 serum cc. continuous	-	83	1.002	0.999–1.004	0.126	-	-	-

LN—lymph node metastasis, M—distant metastasis. Ref—referent. Bold *p*-values were <0.05. In the ICI cohort, due to the small case numbers no Cox analysis could be performed. * TNM was available for 58 patients.

## Data Availability

The data that support the findings of this study are available from the corresponding author upon reasonable request.

## Supplementary Materials

The following are available online at https://www.mdpi.com/article/10.3390/cancers13112548/s1, Figure S1: Overall survival stratified by pretreatment sPD-L1 levels in the subgroup of patients without distant metastasis (M0) (a) and with distant metastasis at the baseline of chemotherapy (M1) (b), Figure S2: Correlation analysis between serum PD-L1 and MMP-7, Table S1: Clinical characteristics in platinum-treated patients, Table S2: Correlation of pretreatment sPD-L1 levels (pg/mL) with clinicopathological parameters in platinum-treated patients. Table S3: Cox univariate OS analysis in treatment subgroups of platinum-treated patients.
