# Supplementary material for: High Serum PD-L1 Levels Are Associated with Poor Survival in Urothelial Cancer Patients Treated with Chemotherapy and Immune Checkpoint Inhibitor Therapy
Source: Cancers (Basel). 2021 May 22;13(11):2548. doi: 10.3390/cancers13112548 (PMC8196869; doi:10.3390/cancers13112548)
Supplement: Supplementary file 1 [file cancers-13-02548-s001.zip › cancers-1170390-supplementary.pdf]

Supplementary Materials

High Serum PD-L1 Levels are Associated with Poor Survival in Urothelial Cancer Patients Treated with Chemotherapy and Immune Checkpoint Inhibitor Therapy

Ulrich Krafft, Csilla Olah, Henning Reis, Claudia Kesch, Christopher Darr, Viktor Grünwald, Stephan Tschirdewahn, Boris Hadaschik, Orsolya Horvath, Istvan Kenessey, Peter Nyirady, Melinda Varadi, Orsolya Modos, Anita Csizmarik and Tibor Szarvas

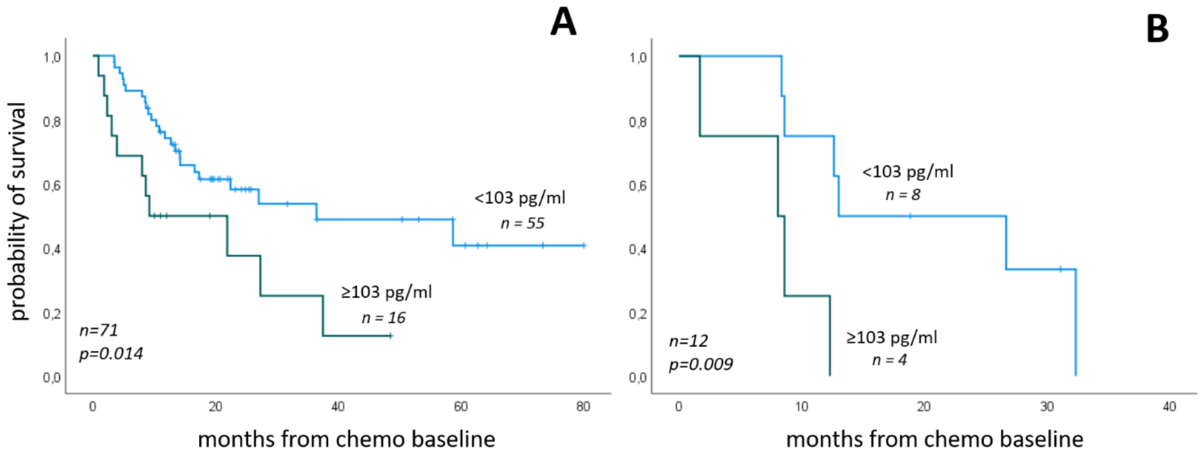

Figure S1. Overall survival stratified by pretreatment sPD-L1 levels in the subgroup of patients without distant metastasis (M0) (a) and with distant metastasis at the baseline of chemotherapy (M1) (b).

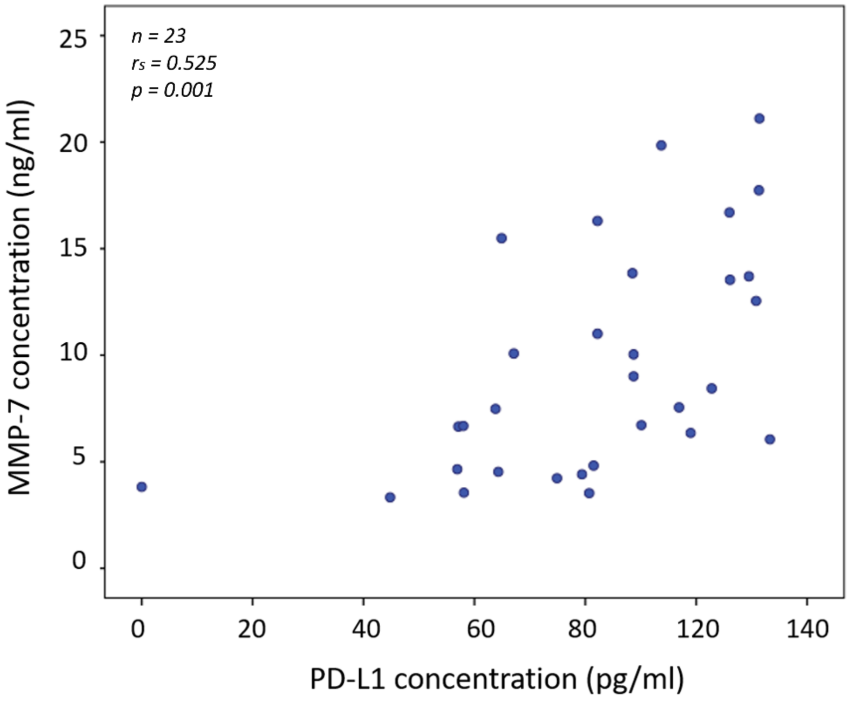

Figure S1. Correlation analysis between serum PD-L1 and MMP-7.

Table S1. Clinical characteristics in platinum-treated patients. ECOG PS- Eastern Cooperative Oncology Group performance status. LN - lymph node metastasis, R - positive surgical margin, RC - cystectomy, Gem/Cis - gemcitabine + cisplatin, Gem/Carbo -gemcitabine + carboplatin, ICI - immune checkpoint inhibitor.

**variables**	**whole chemo cohort**	**neoadjuvant**	**adjuvant**	**palliative**
	n (%)	*n* (%)	*n* (%)	*n* (%)
Total number of patients	83	22	33	28
Age at baseline median [range]	67 [41-81]	68 [48-74]	66 [41-80]	68 [48-81]
Gender				
male	64 (77)	16 (73)	25 (76)	23 (82)
female	19 (33)	6 (27)	8 (24)	5 (18)
ECOG PS at enrollment				
0	48 (58)	8 (36)	27 (82)	13 (46)
1	32 (37)	14 (64)	6 (18)	12 (43)
2	3 (3)	0 (0)	0 (0)	3 (11)
Cystectomy data				
Cystectomy performed	59 (71)	22 (100)	33 (100)	4 (14)
pT0	3 (5)	3 (14)	0 (0)	-
pT1	2 (3)	2 (9)	0 (0)	-
pT2	11 (19)	3 (14)	6 (18)	2 (7)
pT3	30 (51)	10 (45)	19 (58)	1 (4)
pT4	12 (20)	3 (14)	8 (24)	1 (4)
n.a.	1	1	0	0
R	10 (17)	0 (0)	10 (30)	0
LN metastasis at RC	30 (51)	5 (23)	25 (76)	0
Chemo baseline data				
LN at baseline	36 (43)	5 (23)	27 (82)	4 (14)
Distant metastasis at baseline	12 (15)	0 (0)	0 (0)	12 (43)
Soft tissue lesions (lung/liver)	9 (11)	0 (0)	0 (0)	9 (32)
Bone metastasis	3 (3)	0 (0)	0 (0)	3 (11)
Chemotherapy regimen				
Gem/Cis	61 (73)	16 (73)	29 (88)	12 (43)
Gem/Carbo	22 (27)	6 (27)	4 (12)	16 (57)
Atezolizumab	-	-	-	-
Pembrolizumab	-	-	-	-
Number of cycles median (range)	3 (1-9)	3 (1-6)	3 (1-9)	3 (1-6)
single (only one series)	9	1	2	6
Number of patients died (%)	45 (54)	7 (32)	20 (61)	18 (64)
Follow-up time in months median (range)	14 (1-80)	15 (5-48)	19 (2-80)	12 (1-73)
PD-L1 serum levels				
PD-L1 median (range) baseline [pg/ml]	83.0 (0.0 - 781)	93.6 (32.4 -324.0)	80.7 (0.0 - 158.5)	83.6 (25.8 - 780.9)

PD-L1 median (range) 2-3 cycle [pg/ml]	78.5 (0.0 - 273.1)	88.3 (0.0 - 273.1)	76.2 (44.9 - 134.4)	77.9 (38.7 - 261.2)

Table S2. Correlation of pretreatment sPD-L1 levels (pg/ml) with clinicopathological parameters in platinum-treated patients. ECOG—Eastern Cooperative Oncology Group performance status, LN – lymph node metastasis, M – distant metastasis, ICI – immune checkpoint inhibitor. In the ICI cohort, due to the small number of cases no Cox regression analysis could be performed. Bold printed p-values were significant (<0.05).

**variables**	**whole chemo cohort**			**neoadjuvant**			**adjuvant**			**palliative**			**ICI cohort**		
	n	**median (range)**	**P**	***n***	**median (range)**	**P**	***n***	**median (range)**	**P**	***n***	**median (range)**	**P**	***n***	**median (range)**	P
Age															
≤ 65	35	82.2 (0.0-780.9)	0.43	8	93.6 (32.4-324.0)	0.946	16	76.8 (0.0-158.5)	0.815	11	74.9 (25.8-780.9)	0.323	3	86.6 (59.0-169.0)	**-**
> 65	48	84.0 (35.8-243.1)		14	89.7 (46.0-194.9)		17	80.7 (40.5-136.8)		17	85.5 (35.8-243.1)		9	93.3 (25.3-145.0)	
Sex															
Male	64	84.0 (0.0-780.9)	0.435	16	87.0 (32.4-324.0)	0.606	25	80.0 (0.0-136.8)	0.275	23	85.5 (25.8-780.9)	0.697	10	94.2 (59.0-169.0)	-
Female	19	82.2 (40.5-194.9)		6	100.3 (41.9-194.9)		8	91.5 (40.5-158.5)		5	82.2 (48.0-154.8)		2	26.1 (25.3-26.89)	
ECOG															
0	48	80.4 (0.0-158.5)	**0.007**	8	71.2 (32.4 - 111.4)	0.101	27	67.1 (0.0-158.5)	0.1	13	81.0 (25.8-111.3)	0.214	10	91.9 (26.9-169.0)	-
1-2	35	96.2 (35.8-780.9)		14	97.0 (41.9-324.0)		6	91.9 (79.4-136.8)		15	95.9 (35.8-780.9)		2	59.3 (25.3-93.3)	
Stage at cystectomy															
pT0-pT2	16	84.7 (32.4-194.9)	0.741	8	86.9 (32.4-194.9)	**0.001**	6	89.4 (52.3-109.5)	0.692	2	-	-	2	56.8 (26.9-86.6)	-
pT3-pT4	42	80.4 (0.0-324.0)		13	96.2 (41.9-324.0)		27	80.0 (0.0-158.5)		2	-	-	6	97.2 (59.0-169.0)	
LN / M status															
N0/M0	38	87.0 (38.5-324.0)	0.209	17	97.0 (41.9-324.0)	0.322	6	84.7 (67.1-102.2	0.469	15	85.5 (38.5-189.5)	0.818	2	87.1 (80.9-93.3)	-
N+ or M+	45	80.7 (0.0-780.9)		5	91.0 (32.4-114.8)		27	79.4 (0.-158.5)		13	80.7 (25.8-780.9)		10	90.9 (25.3-169.0)	

Table 3. Cox univariate OS analysis in treatment subgroups of platinum-treated patients. ECOG - Eastern Cooperative Oncology Group, LN – lymph node metastasis, M – distant metastasis. Ref – referent. Bold p-values were <0.05. In the ICI cohort, due to the small case numbers no Cox analysis could be performed. Bold printed p-values were significant (<0.05).

**variables**	**whole chemo cohort**				**neodjuvant**				**adjuvant**				**palliative**			
	n	**HR**	**95% CI**	**P**	***n***	**HR**	**95% CI**	**P**	***n***	**HR**	**95% CI**	**P**	***n***	**HR**	**95% CI**	**P**
Age																
≤ 65	35	ref.			8	ref.			16	ref.			11	ref.		
> 65	48	1.297	0.716 - 2.351	0.391	14	0.382	0.071 - 2.042	0.26	17	1.115	0.463 - 2.686	0.808	17	4.165	1.158 - 14.974	**0.029**
Gender																
Male	64	ref.			16	ref.			25	ref.			23	ref.		
Female	19	0.815	0.392 - 1.694	0.584	6	0.344	0.041 - 2.915	0.328	8	1.268	0.458 - 3.110	0.647	5	0.695	0.197 - 2.447	0.571
ECOG																
0	48	ref.			8	ref.			27	ref.			13	ref.		
1-2	35	2.615	1.381 - 4.950	**0.003**	14	2.703	0.490 - 14.913	**0.254**	6	3.032	1.043 - 8.814	**0.042**	15	3.33	1.137 - 9.752	**0.028**
Stage at cystectomy																
T0-T2	14	ref.			8	ref.			6	ref.				ref.		
T3-T4	40	1.135	0.483 - 2.664	0.772	13	2.964	0.345 - 25.484	0.322	27	0.926	0.308 - 1.778	0.89		-	-	-
LN / M status																
N0/M0	38	ref.			17	ref.			6	ref.			15	ref.		
N+ or M+	45	1.914	1.017-3.601	**0.044**	5	1.583	0.289 - 8.682	0.597	27	2.708	0.625 - 11.741	0.183	13	2.182	0.802 - 5.934	0.127
PD-L1 serum cc. / upper 25%																
< 103 pg/ml	63	ref.			16	ref.			27	ref.			20	ref.		
≥ 103 pg/ml	20	1.381	1.120 - 1.703	**0.002**	6	0.746	0.364 - 1.530	0.424	6	1.577	1.134 - 2.192	**0.007**	8	1.51	1.095 - 2.083	**0.012**
PD-L1 serum cc. / median																
< 83 pg/ml	42	ref.			10	ref.			18	ref.			14	ref.		
≥ 83 pg/ml	41	1.989	1.099 - 3.600	**0.023**	12	0.857	0.187 - 3.916	0.842	15	1.684	0.698 - 4.062	0.246	14	4.234	1.537 - 11.667	**0.005**
